# Noninvasive Diagnostic Modalities in an Isolated Case of Cardiac Amyloidosis

**DOI:** 10.7759/cureus.14608

**Published:** 2021-04-21

**Authors:** Jennaire Lewars, Hersh Wazir, Brandon Gordon, Uyi Faluyi, Yousry Girgis

**Affiliations:** 1 Internal Medicine, Saint James School of Medicine, Chicago, USA; 2 Medicine, All Saints University College of Medicine, Toronto, CAN; 3 Medicine, Saint James School of Medicine, Chicago, USA; 4 Medicine, All Saints University School of Medicine, Toronto, CAN; 5 Internal Medicine, Holy Cross Hospital, Chicago, USA

**Keywords:** attr amyloidosis, restrictive cardiomyopathy, amyloid cardiomyopathy, heart failure, ttr, cardiac amyloidosis

## Abstract

Amyloidoses are a family of inherited or acquired disorders characterized by the deposition of insoluble extracellular protein fibrils in various organs and tissues, thereby impairing their function. Amyloidoses are typically misfolded proteins, and on rare occasions, can deposit in the myocardium resulting in an infiltrative/restrictive cardiomyopathy. Restrictive cardiomyopathy is an underdiagnosed cause of congestive heart failure (CHF) with preserved ejection fraction, atrial and ventricular arrhythmias along with conduction defects. In elderly patients, as with this study, cardiac amyloidosis most often results from abnormalities in the liver protein transthyretin (TTR), a thyroxine and retinol-retinol binding complex transporter in blood. Mutated serum TTR results in familial systemic amyloidosis, whereas wild-type TTR results in senile cardiac amyloidosis predominantly seen in elderly males. Scintigraphy, a common non-invasive method used to facilitate early diagnosis of cardiac amyloidosis was the method used in this study. However, the gold standard for definitive diagnosis of cardiac amyloidosis is endomyocardial biopsy (EMB). Besides organ transplant, which is rarely done, therapy for cardiac amyloidosis is mainly aimed at symptomatic and supportive care. Plenty of evidence has shown that the left ventricular ejection fraction (LVEF) in patients with restrictive cardiomyopathy is usually preserved. However, in this study, we review the unique case of an 82-year-old male who was diagnosed with isolated cardiac amyloidosis with severe systolic dysfunction (decreasedLVEF), the methods used to establish the diagnosis, as well as the therapeutic interventions.

## Introduction

Amyloidosis is a term that encompasses a rare group of systemic diseases inclusive of the coalescence and extracellular deposition of insoluble fibrillary proteins known as amyloid in various organs. The disease subtype is based on the type of amyloid protein and the organ that this manifestation predominates.These proteins accumulate in the respective organ, progressively replacing the native tissue parenchyma resulting in eventual organ failure [1].

In the case of cardiac amyloidosis, amyloid deposits within the walls of the heart resulting in a restrictive pattern of cardiomyopathy. The resultant sequelae of cardiac amyloidosis are highlighted by congestive heart failure (CHF), arrhythmias, and heart block resulting in symptoms and presentations attributed to each respective complication. Although cardiac involvement is a common feature of systemic amyloidosis, it is fairly rare in isolation and may only produce subtle clinical findings[2].

In addition to the pertinent historical findings and presentation, diagnostic evaluation of cardiac amyloidosis incorporates the modalities of electrocardiograms, echocardiograms, and cardiac magnetic resonance imaging. However, the gold standard of diagnosis of cardiac amyloidosiscomes from the myocardial biopsy which reveals histological evidence of amyloid material utilizing Congo red stain, with the presence of apple-green birefringence under polarized light conditions [1].

The treatment of cardiac amyloidosis hinges primarily on supportive measures, attenuating the symptoms of heart failure, and slowing the progression of amyloid deposition.Though various forms of cardiac amyloidosis show variable outcomes, the disease process carries a poor prognosis with a high annual mortality rate.

In this case study, we present a case of isolated cardiac amyloidosis in an elderly male patient with a history of hypertension and left ventricular dysfunction; initially presenting as progressive CHF.

## Case presentation

An 82-year-old male patient with a history of hypertension, hyperlipidemia, CHF, and chronic obstructive pulmonary disease (COPD) presented to the emergency department due to worsening progression of CHF and COPD, manifested by shortness of breath and bilateral lower extremity edema with additional concerns of coronary artery disease (CAD) and acute cerebral infarction.The patient endorsed a history of tobacco use up until two weeks prior to presentation but denied any usage of alcohol or illicit drugs and denied any pertinent family history.Echocardiography from one month prior revealed an ejection fraction of 45% to 50% with diastolic dysfunction.

**Figure 1:**
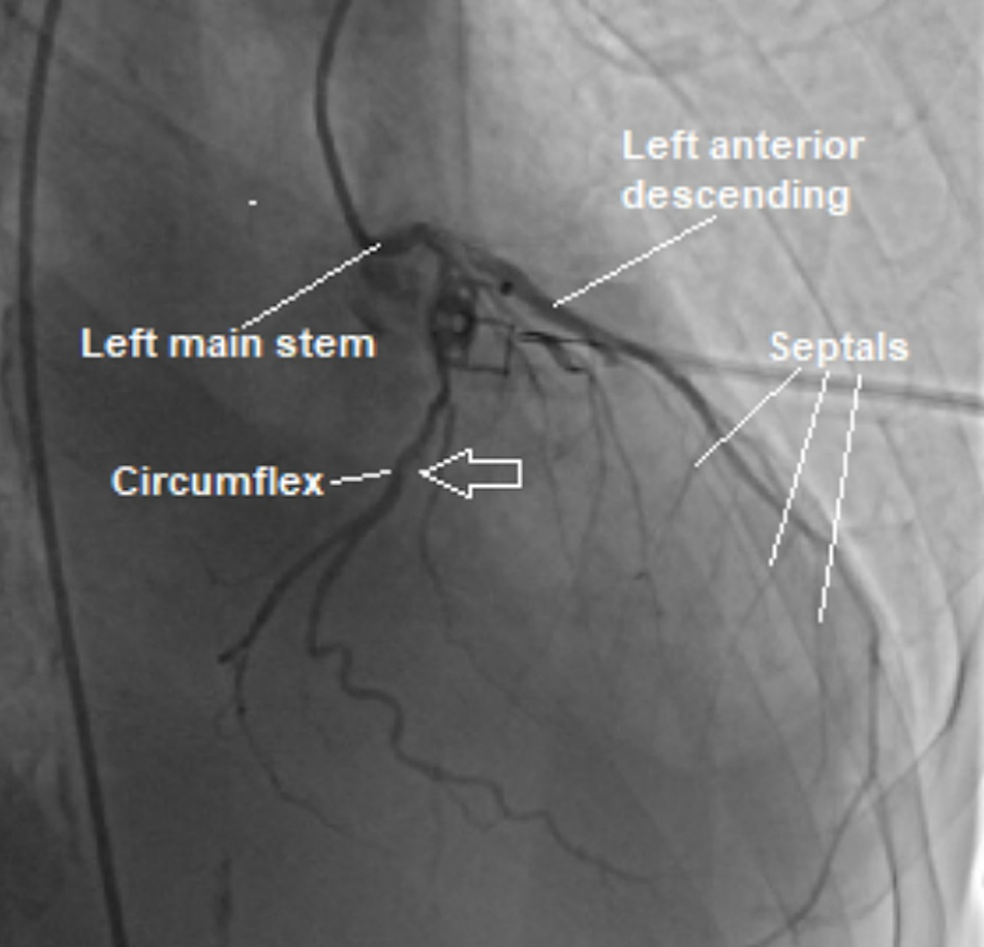
Cardiac angiogram demonstrating stenosis of the left circumflex artery.

**Figure 2:**
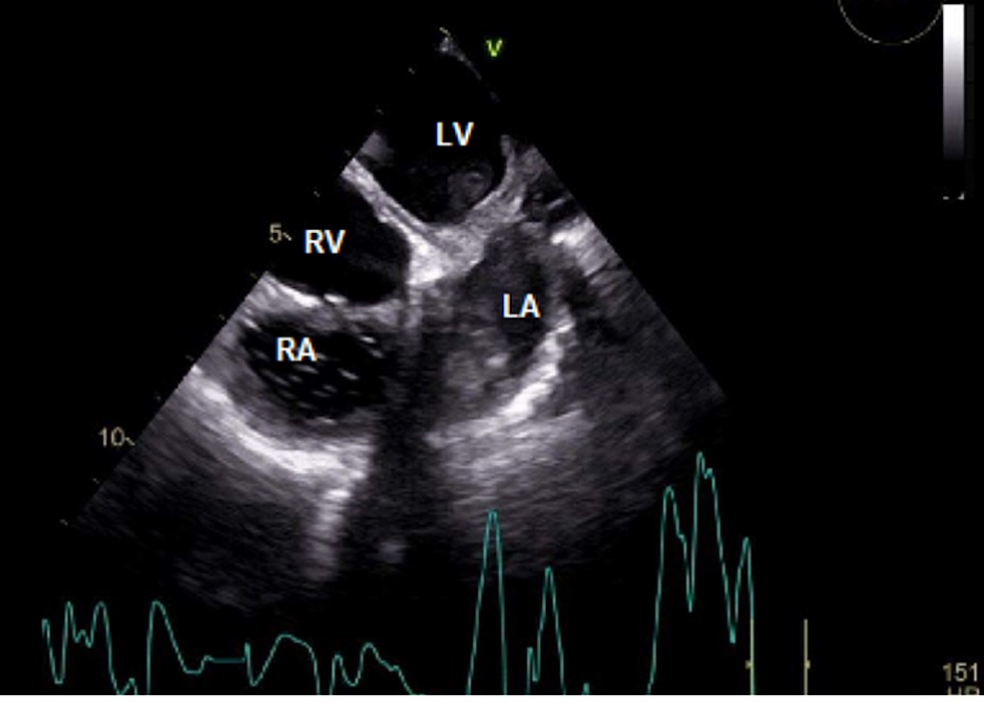
Transesophageal echocardiogram demonstrating left atrial mural thrombus. RA: Right atrium; LA: Left atrium; RV: Right ventricle; LV: Left ventricle

**Figure 3:**
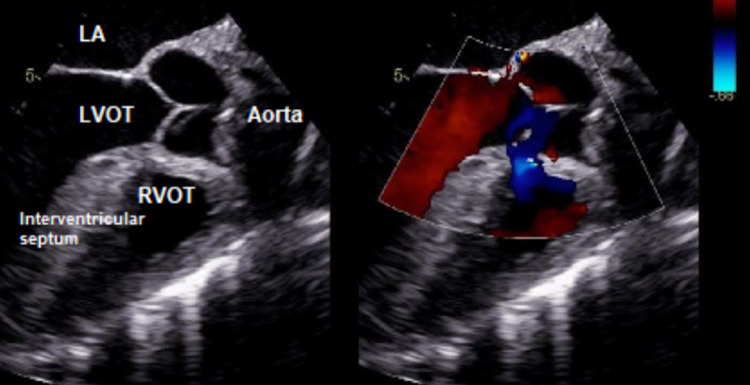
Transesophageal echocardiogram demonstrating ventricular septal hypertrophy. LA: Left atrium; LVOT: Left ventricular outflow tract; RVOT: Right ventricular outflow tract

**Figure 4:**
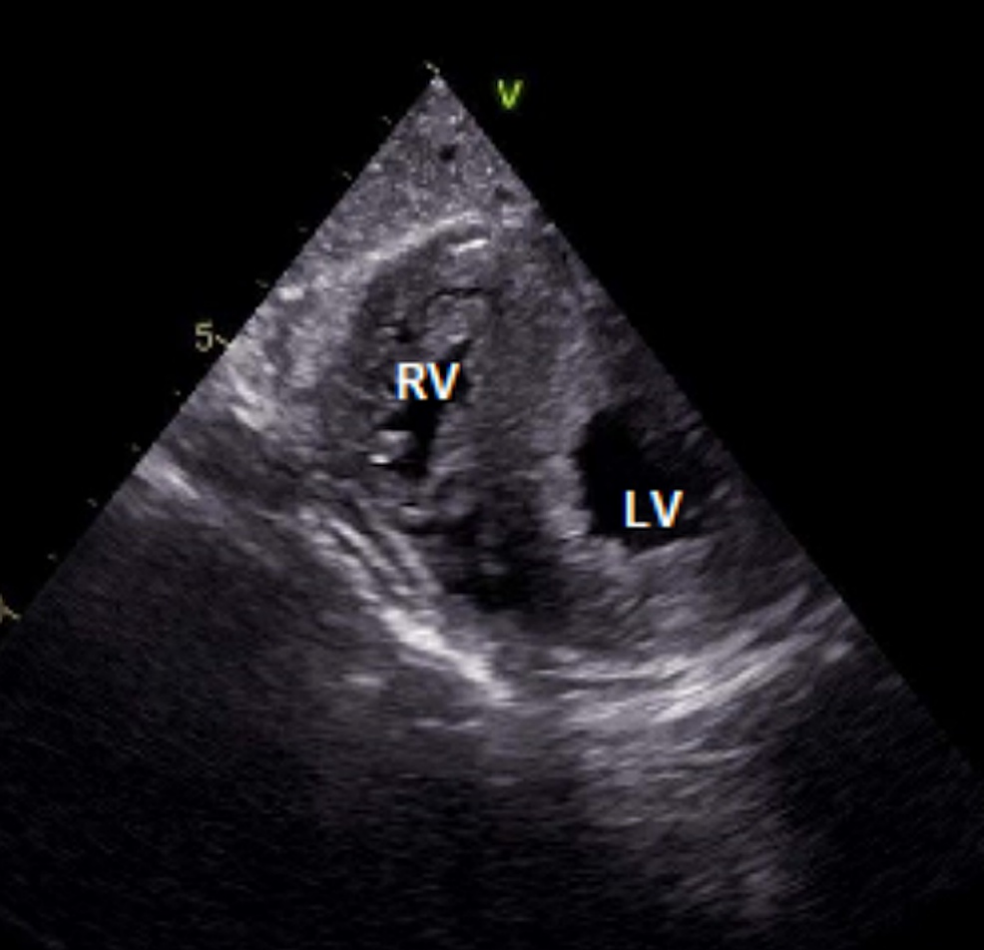
Transesophageal echocardiogram demonstrating ventricular septal hypertrophy from the transverse plane. RV: Right ventricle; LV: Left ventricle

At the time of presentation, physical assessment revealed a systolic ejection murmur heard best at the aorticwith normal S1 and S2 heart sounds, an absence of jugular venous distention with clear lungs auscultated bilaterally.Initial diagnostic assessment included a 12-lead electrocardiogram assessment, obtainment of cardiac markers, a chest X-ray (CXR), transesophageal echocardiogram (TEE) study and nuclear medicine evaluation via technetium pyrophosphate scan. The ECG revealed sinus rhythm with a 1st-degree atrio-ventricular block with occasional pre-ventricular contractions, ST and T wave abnormalities, and evidence suggestive of inferolateral ischemia.CXR revealed mild cardiomegaly and right sided pleural effusion, bilateral lung consolidation, atelectasis, and mild interstitial edema.The pro-brain natriuretic peptide (proBNP) was noted to be 1057, with equivocal troponins.Catheterization presented results of an ejection fraction of 20%-25%, 50% stenosis of the left circumflex artery (as seen in Figure 1), withTEE revealing severe diffuse hypokinesis complicated by a 1.2 x 1.2 cm left atrial thrombus (as seen in Figure 2), a calcified mitral valve leaflet, a reduced left ventricular cavity size with increased wall thickness, ventricular septal hypertrophy, as well as evidence of marked fibrocalcific changes to the aortic root and aortic valve sclerosis (Figures 3-4).Cardiac amyloidosis may be clinically suspected in patients with heart failure with co-existing unexplained left ventricular hypertrophy (LVH) as evidenced in this patient.With the concomitant symptoms of presyncope, syncope, and angina with LVH, suspicion for amyloidosis was heightened.

**Figure 5:**
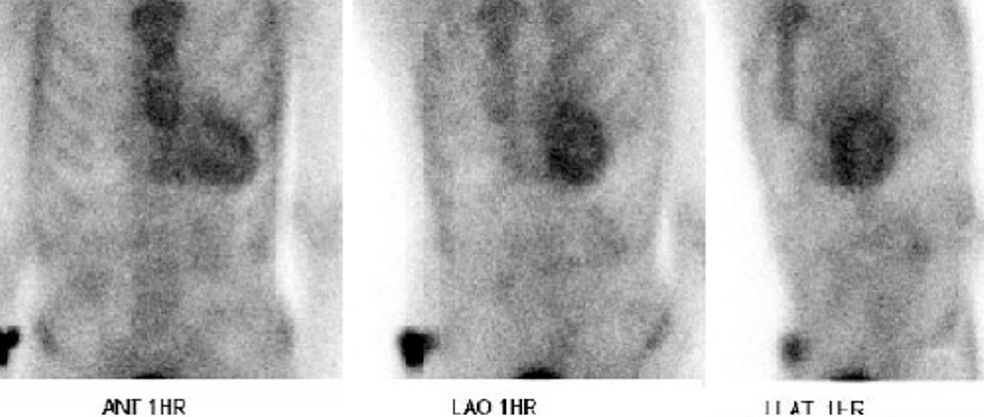
Technetium pyrophosphate scan revealing radial pharmaceutical uptake.

**Figure 6:**
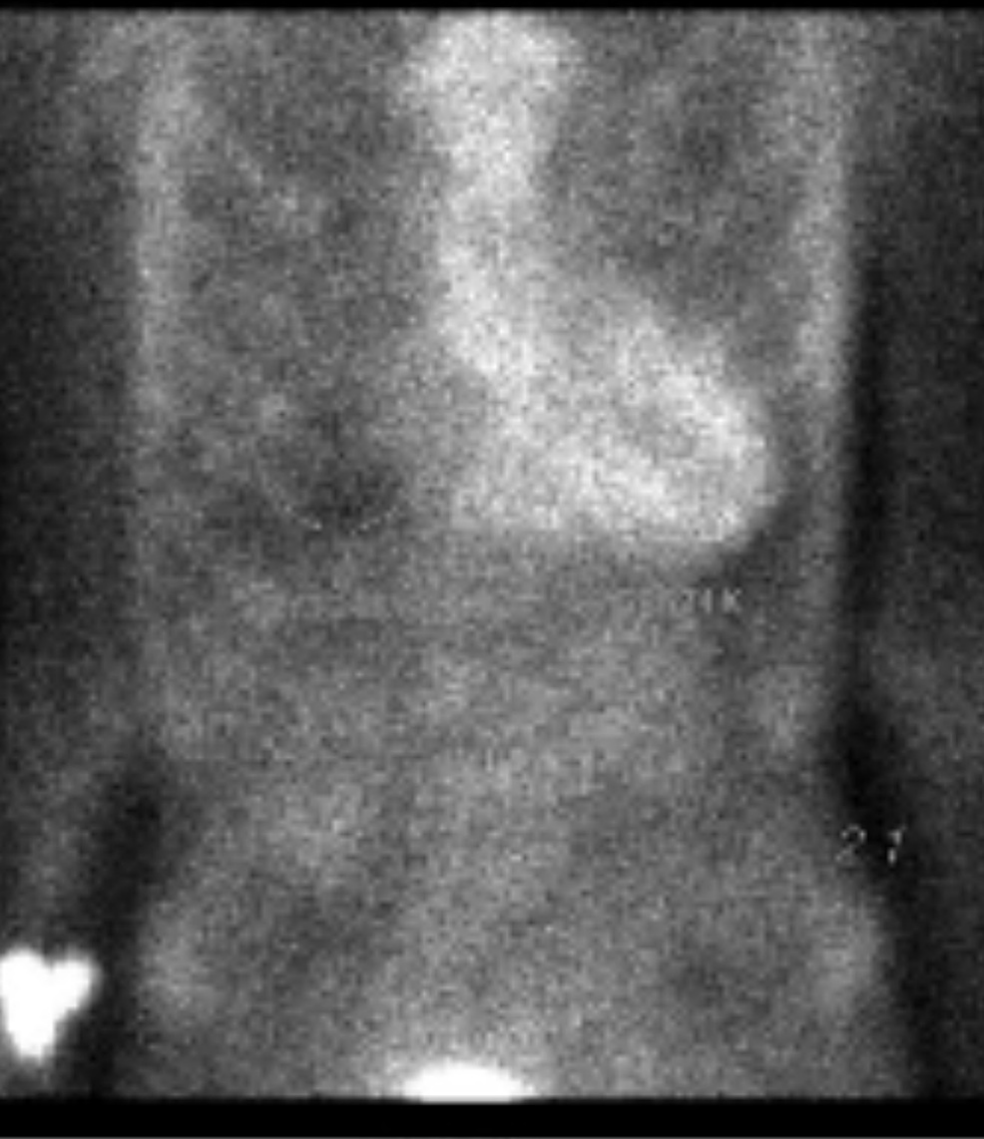
Technetium pyrophosphate scan revealing radioactive pharmaceutical uptake and 2:1 heart/contralateral ratio.

To further assess the differential diagnosis of cardiac amyloid disease, a myriad of tests were performed. Nuclear medicine assessments utilizing 22.5 mCi of technetium pyrophosphate performed in anterior, left anterior oblique, and left lateral projections were undertaken revealing scintigraphic evidence oftransthyretin amyloidosis (ATTR) type cardiac amyloidosis.Upon administration, radial pharmaceutical retention was noted within the myocardium, by which qualitative analysis indicated that myocardial uptake was greater than that of rib uptake (as seen in Figure 5), suggesting grade 3 qualitative amyloidosis with a H/CL (Heart to Contralateral) ratio of 2:1 (ratios: >1.5 are considered positive for the diagnosis of ATTR amyloidosis) (Figure 6).

Inpatient management included utilization of a LifeVest cardioverter defibrillator, and maintenance of euvolemic status via lasix, spironolactone, lisinopril and metoprolol. The COPD exacerbation was managed with ipratropium-bromide/albuterol, montelukast and budesonide. The patient was coumadinized to address the left atrial thrombus. The patient was referred to Cardiology at an outpatient center for subsequent endomyocardial biopsy (EMB) as per hematologist-oncologist recommendations. However, due to the invasiveness of the procedure, the patient elected for a cardiac MRI instead.Cardiac MRI with and without contrast revealed a normal LV size and wall thickness, with a mildly reduced systolic function globally and aleft ventricular ejection fraction (LVEF) of 40%.RV was normal in size with a moderately reduced systolic function.Mild mitral regurgitation was noted.Delayed enhancement imaging for viability was abnormal, with diffuse and hyperenhancement of the myocardium in almost circumferential distribution, particularly in the subendocardium.There was some hyperenhancement of the RV septal wall.According to the overseeing cardiologist, this pattern was highly suggestive of cardiac amyloidosis. A discussion was initiated with the patient regarding the utilization of Tafamidis, an organic compound indicated for the treatment of the cardiomyopathy of wild type or hereditarytransthyretin (TTR)-mediated amyloidosis and an overview of its benefit in TTR amyloid cardiomyopathy.Trial of Tafamidis requires a confirmatory diagnosis of cardiac amyloidosis via cardiac biopsy.However despite elucidating the necessity of the biopsy for the treatment modality and the establishment of his candidacy for its use, the patient once more denied undergoing the biopsy.

## Discussion

Cardiac amyloidosis is a condition resulting from the deposition and accumulation of insoluble fibrillary proteins known as amyloidwithin the extracellular region of the heart [1].There are five primary types of amyloidcontributory to this condition: TTR, light chain (AL), amyloid A (AA), systemic senile, isolated atrial/secondary by which this exceptionally rare condition features one of these proteins in predominance [2].The two most common forms of amyloid implicated in cardiac amyloidosis are AL and ATTR amyloid, with the TTR related form holding two further subtypes being the hereditary (or mutated) subtype, and the senile (non-mutated), senile systemic amyloidosis (SSA) subtype [2].AL amyloidosis is an acquired disease whereby TTR-related forms may hold a genetic predisposition. The diagnostic workup undertaken once cardiac amyloidosis is suspected involves the utilization of characteristic electrocardiogram, echocardiogram, cardiovascular magnetic resonance imaging (CMR), and monoclonal protein isolation findings [1]. A tissue biopsy of the myocardium, historically being the gold standard technique, may be needed in atypical cases to provide confirmatory data.

Findings relative to each form of amyloid vary per respective diagnostic modality.On electrocardiogram, a low QRS voltage and pseudo-infarct pattern is noteworthy. However, studies indicate that the low voltage waveforms predominate in the AL subtype in contrast to the ATTR amyloid subtype, with the pseudo-infarct pattern presenting in both [1].

Due to its phenotypic variability and owing to its increasing prevalence in patients with unexplained LVH [3], the possibility of ATTR should be considered by physicians as a cause of CHF in the elderly. Utilizing echocardiograms, as one of the preliminary means of diagnostic evaluation in CHF, findings relative to cardiac amyloidosis typically include ventricular hypertrophy with a speckling appearance, a diminishment in ventricular chamber size, valve thickening, dilated atria, and a restrictive diastolic physiology producing elevated filling pressures [1]. An international consensus panel regarding amyloid disease notes that an interventricular septal thickness of >12 mm in the absence of hypertensive disease or aortic valve pathology is the diagnostic criterion via echocardiography that substantiates cardiac involvement in patients with known systemic amyloidosis [4].In most cases, a paramount feature demonstrated by a positive test is apical sparing of longitudinal strain. This is a key measure of systolic dysfunction and such longitudinal strain pattern has demonstrated a high sensitivity (93%) and specificity (82%) [5].

Cardiac magnetic resonance imaging produces superior results to echocardiography as it provides a superior degree of myocardial border delineation and provides three-dimension aspects to assessing ventricular volumes, wall thickness and mass [4].Utilizing cardiac magnetic resonance imaging, the key finding is delayed gadolinium enhancement.Gadolinium is an extracellular contrast agent whereby, when administered, in a normal heart the gadolinium is not retained subsequent to its administration.In the occurrence of cardiac amyloidosis, due to the amyloid infiltration, there is an expansion of the extracellular space [4].This is a direct result of the altered fluid kinetics and composition mechanics resulting in an abnormal gadolinium distribution leading to an increased retention of the contrast that produces a contrast enhancement visible on the CMR imaging.The highly characteristic findings indicate the presence of transmural distribution of amyloid deposits supported by a cardiac amyloid load.

When recruiting the modality of myocardial biopsies for diagnosis, results are produced with histological evidence of amyloid material highlighted with Congo red stain.The microbiological examination in the tissue sample of a positive diagnosis will show the presence of amorphous hyaline deposits within the extracellular space, accentuated by apple-green birefringence under polarized light conditions providing confirmation for amyloid material [1].Additionally, cross-beta pleated sheets should be spotted under electron microscopy.However, as with regards to the utilization of myocardial biopsies for confirmation, such an approach is not currently universally required. Instances in which findings from other, less invasive measures provide indicative data are now considered sufficient and are deemed as more conventional approaches. An example of such would be a patient with findings from the aforementioned CMR technique consistent with cardiac amyloidosis, particularly if they have existing systemic AL amyloidosis, which nullifies the need for a myocardial biopsy. Patients with suspected AL amyloidosis have a separate evaluation which briefly consists of abdominal fat pad aspirates, bone marrow biopsy, separate tissue biopsy etc.Fairly recently, a rather remarkable technique involving laser micro-detection with mass spectrometry has been adopted as a gold standard approach for unequivocally identifying amyloid precursor proteins with their respective ensuing amyloid types [6].

The differential diagnosis includes the following possibilities: hypertension-induced LVH, heart failure with preserved ejection fraction (HFpEF), hypertrophic cardiomyopathy and the incredibly rare genetic disorder known as Fabry disease.Since cardiac amyloid deposition is common in elderly patients with heart failure and preserved ejection fraction [7], the diagnosis of amyloidosis may commonly be falsely excluded from differential in an elderly patient who has heart failure with low ejection fraction. As seen in this case study, clinicians should keep an eye for even low ejection fraction when considering amyloidosis.As mentioned, the echocardiogram is useful in its provision of the findings of relative apical sparing of longitudinal strain which is deemed highly sensitive and specific for cardiac amyloidosis. The CMR aids in diagnosis due to resulting delayed gadolinium enhancement. In a patient with a normal heart, the administered gadolinium is not retained by the myocardium as opposed to a heart with amyloid deposits, which serves to narrow the pending differentials.

The increase in N-terminal pro-BNP and worsening reduction of LVEF is significant for clinical progression of heart failure in this patient. The treatment approach in cardiac amyloidosis (chiefly TTR) is targeted at alleviating the symptoms of concomitant heart failure, and slowing or halting the progression and deposition of amyloid [4]. In addition, with reference to TTR cardiac amyloidosis, as TTR is produced via the liver, transplantation of the liver is an option for therapeutic intervention for patients with variant TTR-related amyloid cardiomyopathy [4].

However, with regards to the clinical course of cardiac amyloidosis, the clinical progression depends upon the fibril type, specific mutation, age of onset and the variability of the length of the fibrils involved [1].The prognosis varies between the two forms, with the ATTR form showing a longer median of survival compared to the AL form. Notably, they both carry high annual mortality if untreated, as the condition progresses to intractable heart failure and mortality [2].

## Conclusions

Cardiac amyloidosis has historically been associated with marked morbidity and mortality due to the severe corresponding heart failure. The variety of clinical presentations often leads to misdiagnosis and inappropriate delays in adequate treatment.A high degree of clinical suspicion is required to diagnose, due to its low incidence and low specificity of symptoms. Thus, clues that point toward the diagnosis of this disease are important. The common presenting symptoms are often non-specific to cardiac amyloidosis, but more suggestive of heart failure. Therefore, diagnosis cannot be based solely on one imaging modality; rather, it requires a multidisciplinary approach (biomarkers, imaging, and tissue biopsy) for an early and prompt diagnosis.Also, the diagnosis of amyloidosis should not be excluded from differential in cases of heart failure with low ejection fraction. In the appropriate clinical setting (new-onset heart failure in an elderly male patient) as highlighted, close monitoring and imaging surveillance are recommended, even when initial tests are not pathognomonic for cardiac amyloidosis.
